# Optical actuation of a micromechanical photodiode via the photovoltaic-piezoelectric effect

**DOI:** 10.1038/s41378-021-00249-y

**Published:** 2021-04-14

**Authors:** A. Rampal, R. N. Kleiman

**Affiliations:** 1 Department of Engineering Physics, McMaster University, Hamilton, ON L8S 4L7 Canada; 2 Present Address: CircuitMind Inc, 185 Spadina Avenue, Toronto, ON M5T 2C6 Canada

**Keywords:** Physics, NEMS

## Abstract

Radiation pressure and photothermal forces have been previously used to optically actuate micro/nanomechanical structures fabricated from semiconductor piezoelectric materials such as gallium arsenide (GaAs). In these materials, coupling of the photovoltaic and piezoelectric properties has not been fully explored and leads to a new type of optical actuation that we call the photovoltaic-piezoelectric effect (PVPZ). We demonstrate this effect by electrically measuring, via the direct piezoelectric effect, the optically induced strain in a novel torsional resonator. The micron-scale torsional resonator is fabricated from a lattice-matched single-crystal molecular beam epitaxy (MBE)-grown GaAs photodiode heterostructure. We find that the strain depends on the product of the electro-optic responsivity and piezoelectric constant of GaAs. The photovoltaic-piezoelectric effect has important potential applications, such as in the development of configurable optical circuits, which can be used in neuromorphic photonic chips, processing of big data with deep learning and the development of quantum circuits.

## Introduction

Optical actuation of micro/nanomechanical resonators has been developed across various material platforms and geometries^1–23^. Of particular interest is optical actuation of semiconductor materials^1–15^, given their technological ubiquity in devices such as computers, cell phones, solar cells, lasers, photodiodes, and micro/nanomechanical sensors. One of the main reasons for their ubiquity stems from the capability for high-volume, high-yield manufacturing of micro/nanodevices.

The most common mechanisms used for optical actuation are radiation pressure and photothermal forces. Radiation pressure is a weak force; hence, large optical intensities and/or complex optical setups are required, which limits the circumstances in which these forces can be used. Mechanical structures actuated photothermally can undergo large strains, but their frequency response is limited by the thermal constants of the materials. A further limitation of these mechanisms is that extra steps are required to integrate an electrical transducer to quantify the optically induced strain. The extra steps can come either in the form of additional fabrication steps for integration of a transducer^11,14,15^ or via complicated experimental setups to integrate a transducer external to the device^3,5,7–10,12^. These constraints limit the use of optical actuation to either lab settings or customized applications in commercial settings.

Here, we introduce and demonstrate a new optical actuation mechanism that we call the photovoltaic-piezoelectric (PVPZ) effect. This effect can occur in semiconductor materials that possess both piezoelectric and photovoltaic properties such as those in the III–V and II–VI compound semiconductor families. The piezoelectric property enables coupling between the mechanical and electrical degrees of freedom (DOFs) via the inverse and direct piezoelectric effects. In these effects, an applied voltage results in a mechanical strain, and a mechanical strain results in a charge, respectively. Using both effects together, mechanical strain can not only be electrically induced but also measured. Both of these effects are utilized in research and commercial piezoelectric pressure sensors^24,25^ and accelerometers^26,27^ to measure the strain resulting from external forces. Similarly, resonant quartz tuning forks making use of both the inverse and direct piezoelectric effects are employed in scanning probe microscopy^28,29^ and atomic force microscopy^30,31^ for imaging topographical features of material surfaces. Building on the photovoltaic effect and the inverse piezoelectric effect, the optical actuation is a result of a two-step process in which the incident light induces a photovoltage that causes mechanical strain. Making use of the direct piezoelectric effect, the mechanical strain can be electrically measured directly, hence mitigating the need for extra fabrication steps or complicated experimental setups for the integration of a transducer. The PVPZ effect therefore couples the optical, electrical, and mechanical DOFs of the material. Broadly speaking, the PVPZ effect leads to relatively large forces with a geometry-limited frequency response.

We demonstrate the PVPZ effect using a single-crystal micromechanical device fabricated from a GaAs p-n junction photodiode-solar cell heterostructure, with heterostructure layers added to ensure successful release of the mechanical resonator. GaAs is chosen because it is commonly used to make high-efficiency photodiode-based solar cell structures^32–35^ and has a robust piezoelectric effect^36–38^. Piezoelectric micromechanical devices fabricated from GaAs heterostructures have previously been studied. For example, Masmanidis et al.^36^ demonstrated electrical tuning of the device resonant frequency in a GaAs PIN structure. In their studies, the device was electrically driven, and its mechanical strain was measured using optical interferometry. Okamoto et al.^39^ demonstrated dynamic mechanical coupling between two GaAs bridge structures. In their studies, the devices were electrically driven using the inverse piezoelectric effect, and their motion was detected using the direct piezoelectric effect, as in quartz tuning fork resonators. Our micromechanical structure is a novel planar torsional resonator designed to minimize clamping losses and null out residual photothermal actuation. We chose to use the direct piezoelectric effect to provide a direct measure of deflection due to the seamless and natural integration with the device structure. We also use the indirect piezoelectric effect for electrical actuation of the device for calibration purposes. Our device has a resonant frequency of 76.7 kHz, a room-temperature *Q* of ~6000 and an electro-optic responsivity of 0.178 A/W. We find that the optically induced strain resulting from the PVPZ effect depends on the product of the electro-optic responsivity and the piezoelectric constant and that this strain is linear with optical power, with a nonresonant tip deflection of *δ*_z_ ∼ 11 μm/W.

The demonstration of the PVPZ effect extends the application of semiconductors to the optoelectromechanical domain, addressing the long-standing open issue of providing optical actuation in a semiconductor platform for all-optical computing, processing, and telecommunication applications. The advantages of using semiconductor materials are the ability to make use of conventional batch fabrication methods and the opportunity to leverage mature highly integrated on-chip optical and electronic functionalities.

## Results and discussion

**Fig. 1:**
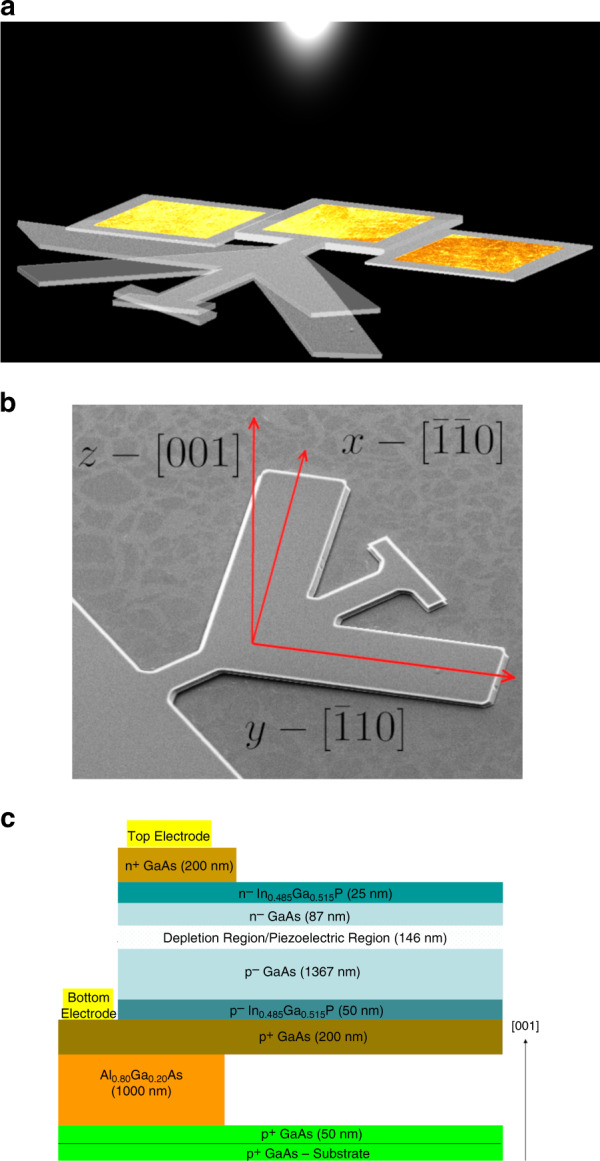
Design of the photodiode torsional resonator. **a** Fundamental undeformed torsional mode shape (semitransparent) and deformed mode shape of the resonator under optical illumination. The illustrated mode shape is determined from simulations (as shown in Supplementary Section V). The resonator is composed of a central spine terminated on the top by a rectangular pad and at the bottom by an anchor, where three contact pads are situated. Protruding from the spine at ±45° are two tines. The electrodes on the anchor facilitate measurement of the piezoelectric current. Above-bandgap light is modulated near the resonant frequency of the resonator, exciting the torsional mode via the PVPZ effect. **b** SEM image of a fabricated torsional resonator on a [001] GaAs wafer. The spine is parallel to the [100]-direction (not marked). The directions of the tines are chosen to exploit the antisymmetry in the III–V piezoelectric properties, resulting in antisymmetric flexural deformation. **c** Cross-section (not to scale) of the MBE-grown photodiode heterostructure. The section shown is the released structure, i.e., the Al_0.8_Ga_0.2_As layer remains in place at the anchor and is removed below the spine and tines. The depletion region is intentionally placed above the neutral plane to enable unimorph-type actuation. The arrow indicates the crystal orientation. The top and bottom electrodes contacting the n^+^ and p^+^ GaAs layers are used to measure the piezoelectric current resulting from the induced strain. The electrodes can also be used to electrically drive the resonator. The center contact pad shown in Fig. 1a connects to the device top electrode, while the two adjacent contact pads connect to the device bottom electrode

We experimentally demonstrate the PVPZ effect by optically actuating a novel micromechanical structure, a planar torsional resonator (Fig. 1a), and electrically measuring the resulting optically induced strain near resonance via the direct piezoelectric effect. The torsional resonator is fabricated from an epitaxially grown GaAs photodiode heterostructure (Supplementary Section I). The torsional deformation arises from the antisymmetric flexural bending of the two diagonally oriented tines that comprise the structure. The driving force for this antisymmetric bending is the result of (a) using the antisymmetric piezoelectric property of GaAs in the plane of the device and (b) designing the photodiode heterostructure with vertical asymmetry about the neutral plane to resemble a unimorph structure. The former can be understood by considering the piezoelectric matrix for a [001]-oriented wafer of GaAs, a III–V semiconductor having a zincblende crystal structure^37^:

1 $$d_{{\mathrm{ij}}} = \frac{{d_{14}}}{2}\begin{array}{*{20}{c}} {\left[ {\begin{array}{*{20}{c}} 0 & 0 & { - \beta } \\ 0 & 0 & \beta \\ 0 & 0 & 0 \\ {2\alpha } & {2\beta } & 0 \\ { - 2\beta } & {2\alpha } & 0 \\ 0 & 0 & 0 \end{array}} \right]} \end{array}$$

where *d*_ij_ are the piezoelectric coefficients, $\beta = {\mathrm{cos}}(2\varphi )$, $\alpha = {\mathrm{sin}}(2\varphi )$ and *φ* is the rotation angle about the [001]-direction with respect to the $\left[ {{\bar 1}{\bar 1}0} \right]$-direction, *φ* = 0. For GaAs, *d*_14_ = 1.345 pm/V. To make use of the planar antisymmetry, the two tines of the torsional resonator are aligned parallel to the two orthogonally oriented [110]-directions (Fig. 1b). According to this matrix, *d*_13_ and *d*_23_ are equal in magnitude but opposite in sign; hence, an electric field applied in the [001]-direction results in equal but opposite longitudinal strains, *ε*, in the $\left[ {{\bar 1}{\bar 1}0} \right]$- and $\left[ {\bar 110} \right]$-directions, where $\varepsilon _1 = d_{13}E_3$ and $\varepsilon _2 = d_{23}E_3$, respectively. The flexural bending of the tines, required for driving and detecting, is achieved by designing the GaAs heterostructure such that the piezoelectric layer is offset from the neutral surface of the tines, in this case near the upper surface of the device (Fig. 1c). The depletion region of the p-n junction of the GaAs photodiode responds similarly to a dielectric material, so an applied voltage leads to a piezoelectric stress. The n- and p-type layers are sufficiently doped to serve as equipotential surfaces for device actuation without the need for metal contact layers that would be opaque to optical illumination. The heterostructure is grown by molecular beam epitaxy (MBE) on a p^+^ GaAs substrate and consists of a 1.0-μm-thick Al_0.8_Ga_0.2_As sacrificial etch layer followed by a 2.075-μm-thick photodiode structure resembling a GaAs solar cell. The doping concentrations and thicknesses of the individual layers (Supplementary Section III) are chosen to achieve successful fabrication of the torsional resonator^40^ and to maximize both the photocurrent and piezoelectric current. The torsional resonator design is chosen to null out, to a first approximation, the driving force from photothermal effects because uniform illumination would result in symmetric photothermal bending of the tines. The PVPZ effect is not unique to this geometry and could be demonstrated and utilized in simpler cantilever structures since the use of both *d*_13_ and *d*_23_ is not required.

**Fig. 2:**
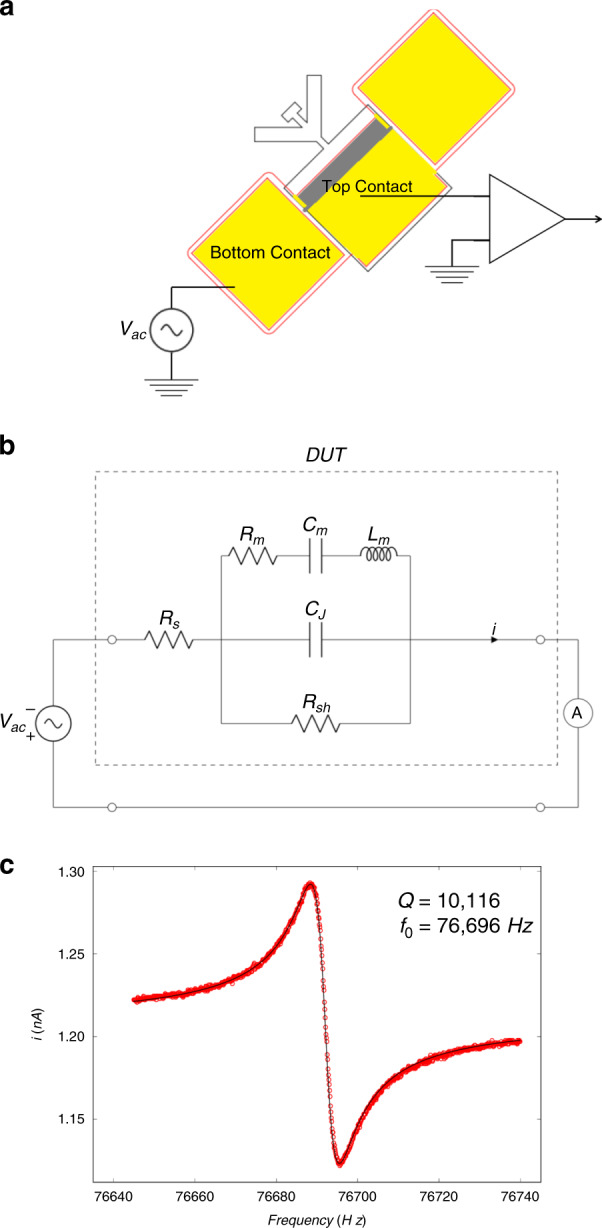
Electrical actuation of the photodiode torsional resonator. **a** Experimental setup for electrically inducing piezoelectric strain and measuring piezoelectric charge. A function generator, represented as *V*_ac_, supplies the AC signal required to drive the resonator near its resonant frequency. The piezoelectric current is first preamplified and then measured using an SR844 Lock-In Amplifier. The resonator and the preamplifier are placed in a vacuum chamber at a pressure of ~2 mTorr. **b** Equivalent circuit of the modified BVD model of a piezoelectric resonator arranged for standard two-terminal drive and detection. The motional components, *R*_m_, *C*_m_ and *L*_m_, are the electrical analogs of a damped harmonic oscillator representing the mechanical dissipation, spring constant, and mass of the resonator, respectively. *C*_J_ is the junction capacitance or geometric capacitance of the device, *R*_sh_ is primarily due to the shunt resistance of the diode, *R*_s_ is dominated by the series resistance of the metal-semiconductor contacts, and *i* is the piezoelectric current. The dashed line encompasses the equivalent circuit of the resonator or device under test (DUT). **c** Frequency response of the measured RMS current with a drive voltage of 66 μV_rms_. The solid line is a fit to the data using equation (S4) derived for the circuit shown in Fig. 2b, and the fitted parameters are given in Supplementary Section VIII. The resonator dimensions for the tine, spine, and pad are 80 × 30 μm, 102.5 × 10 μm, and 40 × 15 μm, respectively, with a total released device thickness of 1.875 μm

In the first set of measurements, we actuate the resonator structure electrically by making use of the inverse piezoelectric effect and measure its motion using the current from the direct piezoelectric effect, providing direct characterization of the device and its piezoelectric and mechanical properties. This permits us to determine the electromechanical device properties entirely in the electric domain using traditional analysis methods^41^. The electromechanical transduction is characterized by applying an AC voltage at frequencies near the resonant frequency (Fig. 2a) across the device and fitting the measured electrical response to the modified Butterworth-van Dyke (BVD) model (Fig. 2b). The measured response (Fig. 2c) agrees very well with the modified BVD model. Using the definitions $\omega _0^2 = 1/\left( {L_{\mathrm{m}}C_{\mathrm{m}}} \right)$and $Q = 1/\left( {\omega _0R_{\mathrm{m}}C_{\mathrm{m}}} \right)$, the resonator is found to have a *Q* of 10,116 and a resonant frequency of $f_0 = \omega _0/2\pi = 76,696$ Hz, which are consistent with expectations from finite element simulations^40^. The resonator dimensions for the tine, spine, and pad are 80 × 30 μm, 102.5 × 10 μm, and 40 × 15 μm, respectively, with a total released device thickness of 1.875 μm. All measurements are performed at room temperature. The high value of *Q* at room temperature is expected because the resonator is designed such that the torsional mode has minimal clamping losses resulting from it being supported at the nodal point^42,43^. The motional capacitance *C*_m_ can be analytically related to the mechanical mode, device geometry, and piezoelectric coefficient (Supplementary Section VII). The junction capacitance, *C*_J_, is dominated by the contact pad, which is much larger in area than the device itself. As a result, the background (or feedthrough) current from *C*_J_ is large, which leads to a large offset in the current, as shown in Fig. 2c.

**Fig. 3:**
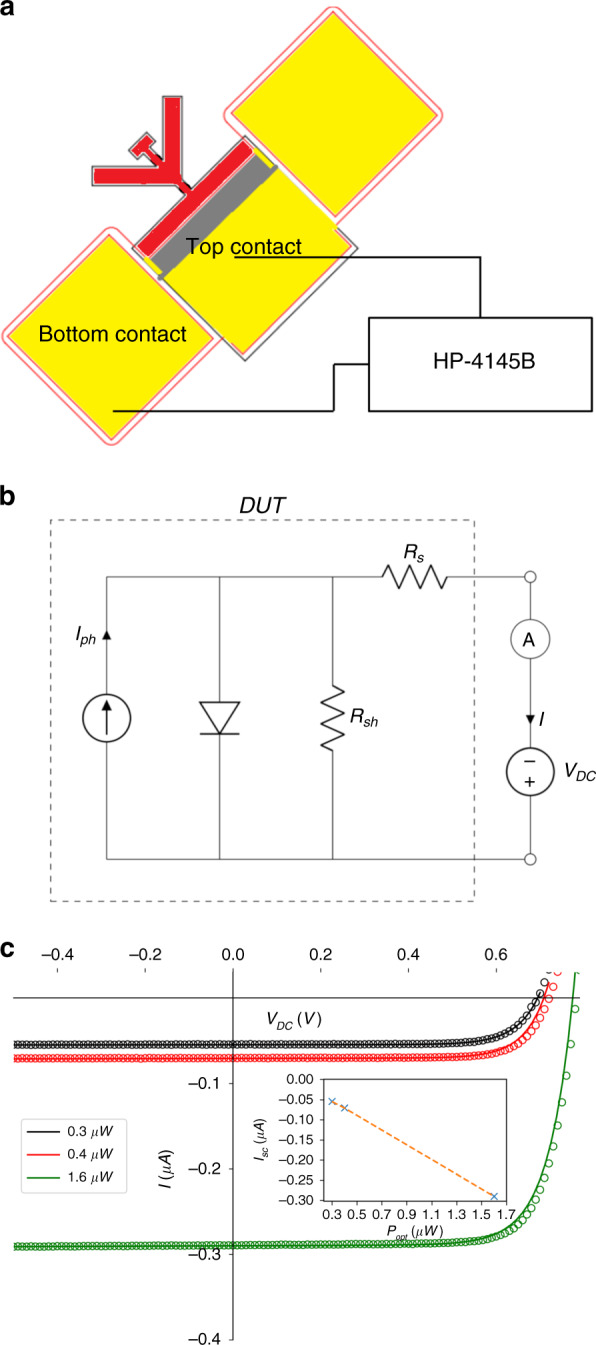
DC optical responsivity of the photodiode. **a** Experimental setup for measuring I–V curves. The photodiode is illuminated by a DigiKey model TSGH510-ND infrared LED, and an HP-4145B semiconductor parameter analyzer measures the generated photocurrent and applies a DC voltage. The illuminated section of the photodiode is shown in red and illustrates homogeneous illumination. Unlike the resonance measurements, the I–V curves are measured at ambient pressure. **b** Equivalent circuit of the single-diode DC PV model. The photocurrent, *I*, from the photodiode is measured at the output terminals as a function of an externally applied DC voltage, *V*_DC_, where *I*_ph_ is the incident photocurrent. The dashed line encompasses the equivalent circuit of the photodiode or DUT. **c** I–V curves of the illuminated photodiode. The solid lines are a fit to the data using equation (S8) derived from the circuit shown in Fig. 3b, and the fitted parameters are given in Supplementary Section IX. The expected linearity of the short-circuit current *I*_sc_ with the incident optical power is shown in the inset

In a second set of measurements, we calibrate the optical response of the photodiode using a DC voltage source and an infrared LED (Fig. 3a) with a peak wavelength of 830 nm and a photon energy just higher than the bandgap of GaAs. The electro-optic responsivity, *R*_resp_, and efficiency of the photodiode in converting optical to electrical power are determined by fitting the measured photocurrent, *I*, to the single-diode DC photovoltaic (PV) model^44^ shown in Fig. 3b and described by Eq. (S8). The measured photodiode I–V characteristics (Fig. 3c) agree well with this model, and the photodiode is found to have *R*_resp_ = 0.178 A/W with an efficiency of approximately 11.5%. The responsivity is calculated as the ratio of the short-circuit current, *I*_sc_, to the incident optical power, *P*_opt_. The short-circuit current is the current measured at *V*_DC_ = 0, which is closely equivalent to the incident photocurrent, *I*_ph_. The efficiency of the photodiode is calculated as the ratio of electrical power at the maximum power point (The maximum power point is defined as a point on the curves in Fig. 3b at which the product of the photocurrent and photovoltage is a maximum) to the incident optical power. In the optical experiments, the LED is placed ~0.5 cm from the device such that the device is homogenously illuminated. Experimental details on the measured optical powers are given in Supplementary Section XI.

**Fig. 4:**
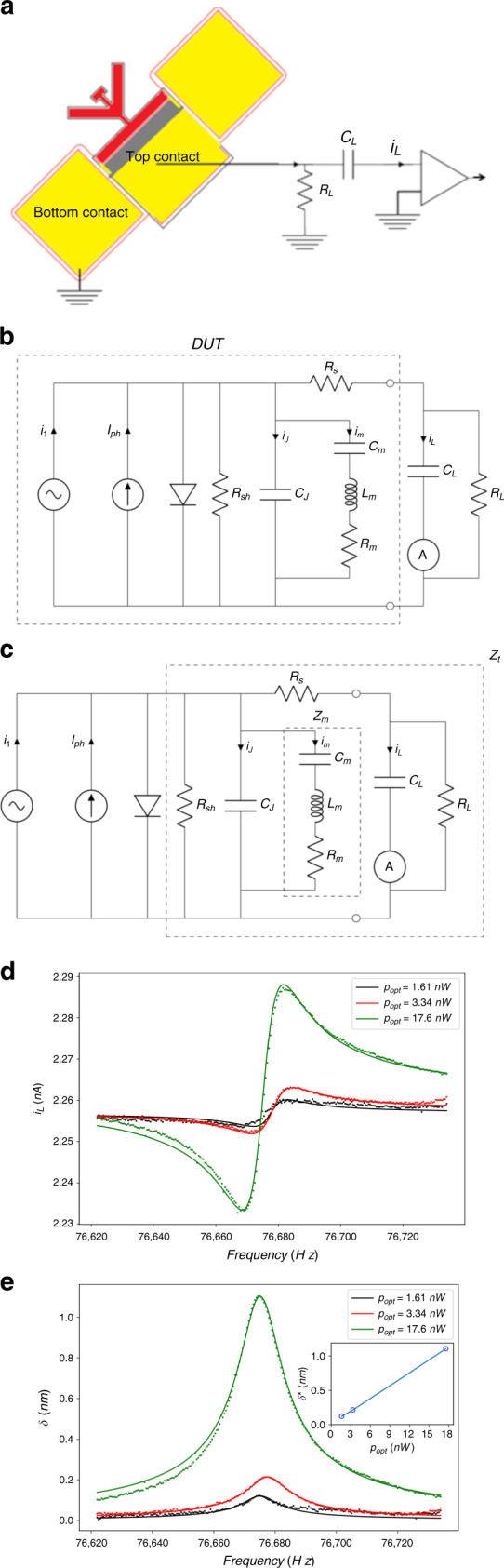
Optical actuation of the photodiode torsional resonator. **a** Experimental setup for measuring the PVPZ effect. The photodiode resonator, optical source, bias T, and preamplifier are placed in a vacuum chamber at a pressure of ~2 mTorr. Similar to the DC measurements, the illuminated section of the resonator is shown in red and illustrates homogeneous illumination. The same 830-nm LED used to characterize the DC efficiency and responsivity is also used here. The bias T is composed of the load capacitance and resistance (*C*_L_ and *R*_L_), and *i*_L_ is the measured current. **b** PVPZ equivalent circuit. To the left of the diode are the AC and DC photocurrents (*i*_1_ and *I*_ph_) photoinduced by the AC and DC optical powers (*p*_opt_ and *P*_opt_) of the LED. To the immediate right of the diode is the BVD circuit similar to Fig. 2b. The load branch is composed of the bias T and is placed at the output terminal or the top electrode of the photodiode. Together, these components establish a DC bias for the PV cell and impedance matching between the device and the measurement circuit. The dashed line encompasses the equivalent circuit of the photovoltaic-piezoelectric resonator or DUT. **c** Same circuit as in Fig. 4b, with the components comprising the total impedance, *Z*_T_, and motional impedance, *Z*_m_, indicated in dashed lines. **d** Frequency response of the measured *i*_L_ for *P*_opt_ of 304 nW and *p*_opt_ of 1.61, 3.34, and 17.6 nW. The solid lines are fits to the data using Eq. (3) derived from Fig. 4c. Equation (3) depends on 4 parameters [*i*_1_*, C*_*m*_, *Q*, and *ω*_*o*_] related to the device and the drive/detection parameters. All four parameters are independently determined through the fits, and the parameters are provided in Supplementary Section X. The values are in reasonable agreement with those determined from purely electrical measurements. The measured currents for the *p*_opt_ values of 1.61 and 3.34 nW have been shifted up so that their background currents align with the background current for *p*_opt_ = 17.6 nW. The fitted *Q* values are 6875, 5825, and 5625 for *p*_opt_ values of 1.61, 3.34, and 17.6 nW, respectively. **e** RMS deflection of the tines due to the PVPZ effect the three different optical power levels. The linearity of the maximum deflection with the drive power is shown in the inset as predicted by Eq. (7)

In a third set of measurements, we actuate the resonator structure optically by making use of the photovoltaic effect in conjunction with the piezoelectric effect and measure its motion using the current from the direct piezoelectric effect. To illustrate the PVPZ effect and hence demonstrate the coupling among the optical, electrical and mechanical DOFs, we modulate the output of the LED sinusoidally and vary the modulation frequency. Figure 4a shows the experimental setup for measuring the PVPZ effect. Since the photovoltaic cell is in parallel with the mechanical resonator by construction, the electrical detection of the mechanical actuation must be established differently than that shown in Figs. 2b or 3b. The equivalent circuit including the actuation and measurement components is shown in Fig. 4b. We set up a parallel RC load branch at the output terminals of the optically driven resonator and measure the current *i*_L_ through *C*_L_. The optically driven resonator circuit is highly nonlinear due to the diode properties, with a general expression for *i*_L_ given in Rampal^40^ for different load conditions. The expression is strongly dependent on the DC bias point, which in this case is determined primarily by *R*_L_. When *R*_L_ < *V*_oc_*/I*_sc_, the device is in the current-dominated regime of the photodiode^40^, where changes in photocurrent, *i*_1_, are directly related to changes in optical power, as in the DC case, i.e., $i_1 = R_{{\mathrm{resp}}}p_{{\mathrm{opt}}}$ and $I_{{\mathrm{ph}}} = R_{{\mathrm{resp}}}P_{{\mathrm{opt}}}$ for the respective AC and DC cases. The value of *R*_L_ must also be chosen so that its impedance is larger than the impedance near resonance of the capacitive components, $C_{\mathrm{T}} = C_{\mathrm{J}} + C_{\mathrm{L}}$, i.e., *ωR*_L_*C*_T_ > 1, to maintain a sufficiently high bandwidth to detect the resonant device operation. Based on these considerations, *R*_L_ and *I*_sc_ should be chosen such that 1/(*ω*_0_*C*_T_) < *R*_L_ < *V*_oc_*/I*_sc_, or 11 kΩ < *R*_L_ < 11 MΩ for our device, and we use a value of *R*_*L*_ = 1.0 MΩ for our measurements. Under these circumstances, we can derive an expression for the complex current, *i*_L_, through the load capacitor – a measurable quantity – by analysis of the equivalent circuit in Fig. 4c:

2 $$i_{\mathrm{L}}(\omega ) = i_1j\omega C_{\mathrm{L}}Z_{\mathrm{t}}$$

3 $$|i_{\mathrm{L}}(\omega )| = i_1\frac{{\left( {a - 1} \right)\sqrt {Q^2\left( {\omega _0^2 - \omega ^2} \right)^2 + \left( {\omega \omega _0} \right)^2} }}{{\sqrt {Q^2\left( {\omega _0^2\frac{{C_{\mathrm{m}}}}{{C_{\mathrm{J}}}} + a\left( {\omega _0^2 - \omega ^2} \right)} \right)^2 + \left( {a\omega \omega _0} \right)^2} }}$$

where *Z*_t_ is the total impedance in parallel with the photodiode, as indicated by the dashed lines in Fig. 4a, and $a \equiv 1 + C_{\mathrm{L}}/C_{\mathrm{J}}$. Circuit analysis shows that the swing in *i*_L_ is maximum when *C*_L_ *=* *C*_J_ or when *a* *=* 2. In our experiments, *C*_L_ = 144 pF and *C*_J_ = 55 pF, resulting in *a* ~3.62. The device is designed such that *R*_s_ is low enough that we can consider it a short circuit and that both *R*_sh_ and *R*_L_ are high enough that they can be considered open circuits for the purpose of AC analysis at frequencies near resonance. To verify the PVPZ effect based on the coupling among the optical, electrical and mechanical DOFs, we drive the resonator optically by modulating the LED at frequencies near the resonant frequency of the device. The measured response (Fig. 4d) agrees well with expectations from the PVPZ circuit (Fig. 4c). The measured resonant frequency is found to be close to the electrically driven case (Fig. 2c), while *Q* is reduced to ~6100, most likely due to aging of the resonator resulting from lack of sidewall passivation. Similar to the feedthrough current in Fig. 2c, the feedthrough current in Fig. 4d is due to parallel paths through *C*_L_ and *C*_J_.

As previously described, it is a good approximation to assume that the motion of the resonator in its torsional mode can be modeled by considering the deflection of the tines as the deflection of cantilevers. In this approximation, we can determine the complex deflection of the cantilever tips as given by^45^:

4 $$\delta _{\mathrm{z}}(\omega ) = - 3\left( {\frac{L}{t}} \right)^2\left( {\frac{{2x_{\mathrm{c}}}}{t}} \right)d_{23}V_{\mathrm{C}} = - 3\left( {\frac{L}{t}} \right)^2\left( {\frac{{2x_{\mathrm{c}}}}{t}} \right)d_{23}\frac{{i_{\mathrm{m}}}}{{j\omega C_{\mathrm{m}}}}$$

where *δ*_z_ is the deflection of the tip, $x_{\mathrm{c}}$ is the distance from the center of the beam to the center of the p-n junction (here, $x_{\mathrm{c}}$ = 752.5 nm), *i*_m_ is the current through the motional arm (*R*_m_, *L*_m_, *C*_m_), *L* and *t* are the length and thickness of the tines and *V*_C_ is the voltage across *C*_m_. Using analysis of the PVPZ circuit (Fig. 4c), we find that the complex deflection is given by:

5 $$\delta _{\mathrm{z}}(\omega ) = - 3\left( {\frac{L}{t}} \right)^2\left( {\frac{{2x_{\mathrm{c}}}}{t}} \right)d_{23}\frac{{i_1 - i_{\mathrm{J}} - i_{\mathrm{L}}}}{{j\omega C_{\mathrm{m}}}} = - 3\left( {\frac{L}{t}} \right)^2\left( {\frac{{2x_{\mathrm{c}}}}{t}} \right)d_{23}\frac{{i_1 - i_{\mathrm{L}}\frac{a}{{\left( {a - 1} \right)}}}}{{j\omega C_{\mathrm{m}}}}$$

which can be used to transform the measured *i*_L_ to a determination of *δ*_z_ (Fig. 4e), based on the fitted values from Supplementary Section X. Figure 4e illustrates a clear resonance (with no antiresonance), as would be expected for a driven simple harmonic oscillator. The peak magnitude is ~1.1 nm RMS for an AC optical modulation of only 17.6 nW, corresponding to a nonresonant (*Q* = 1) actuation of $\delta _{\mathrm{z}}\sim 11\upmu {\mathrm{m}}/{\mathrm{W}}$.

Starting with Eq. (4) and using $i_{\mathrm{m}}Z_{\mathrm{m}} = i_1Z_{\mathrm{t}}$, where *Z*_m_ is the impedance of the motional arm of Fig. 4c, the expression for the bending of the cantilever is given directly in terms of optical parameters, with no reference to electrical currents, by:

6 $$|\delta _{\mathrm{z}}(\omega )| = 3\left( {\frac{L}{t}} \right)^2\left( {\frac{{2x_{\mathrm{c}}}}{t}} \right)d_{23}R_{{\mathrm{resp}}}p_{{\mathrm{opt}}}\frac{{\omega _0^2Q}}{{\omega C_{\mathrm{J}}}}\frac{1}{{\sqrt {Q^2\left( {\omega _0^2\frac{{C_{\mathrm{m}}}}{{C_{\mathrm{J}}}} + a\left( {\omega _0^2 - \omega ^2} \right)} \right)^2 + \left( {a\omega \omega _0} \right)^2} }}$$

where we have substituted $i_1 = R_{{\mathrm{resp}}}p_{{\mathrm{opt}}}$. Since *C*_m_ « *C*_J_ and *Q* » 1, the maximum amplitude, or resonant amplitude, is given by:

7 $$|\delta _{\mathrm{z}}(\omega _{{\mathrm{max}}})| = 3\left( {\frac{L}{t}} \right)^2\left( {\frac{{2x_{\mathrm{c}}}}{t}} \right)d_{23}R_{{\mathrm{resp}}}p_{{\mathrm{opt}}}Q\frac{1}{{\omega _0C_{\mathrm{T}}}}$$

where *ω*_max_ is the resonant frequency. From Eqs. (6) and (7), we can see clearly that the magnitude of *δ*_z_ depends on the product of the optoelectrical material property, *R*_resp_, and the piezoelectric constant, *d*_*23*_, and a proportional and linear change in *δ*_z_ with *p*_opt_ is predicted, as shown in the inset of Fig. 4e. We also see that the mechanical motion is described by a conventional resonant shape without the feedthrough terms present due to electrical detection.

While we have incorporated electrical connections to verify circuit parameters and detect the resonator deflection, this is not required for optical actuation using the PVPZ effect. However, for maximum amplitude, the device needs to be biased at a sufficiently low voltage such that *R*_L_ < *V*_oc_*/I*_sc_; therefore, a load resistance is still required, which could be incorporated into the device structure. While the load capacitance can be eliminated (i.e., *C*_L_ = 0, leading to *a* = 1), *ω*_*0*_*R*_L_*C*_J_ > 1 is still required. By eliminating the contact pad, *C*_J_ can be substantially reduced; however, this makes the latter criterion more difficult to achieve. By choosing *R*_L_ ~ 1/(*ω*_0_*C*_J_) and *I*_sc_ *~* *V*_oc_*/R*_L_, both criteria are just met, and with full modulation and the p-n junction placed close to the device surface, Eq. (7) simplifies to $|\delta _{\mathrm{z}}\left( {\omega _{{\mathrm{max}}}} \right)| \approx \frac{3}{{\sqrt 2 }}\left( {\frac{L}{t}} \right)^2d_{23}V_{{\mathrm{oc}}}Q$, which is the maximum amplitude possible via PVPZ optical actuation.

In measurements (not shown), we observe no resonance within our measurement resolution in the vicinity of the fundamental symmetric mode, expected at 26,550 Hz, confirming the nulling of symmetric actuation and detection. We also fabricate control devices with the same geometry but with the tines of the torsional resonator aligned parallel to the [100]- and [010]-directions, which is expected to lead to no PVPZ effect. The results (not shown) indicate no deflection within our measurement resolution, indicating full rejection of the torsional mode and supporting full rejection of symmetric modes in the original geometry.

Historically, the measurement of subtle optically driven forces, such as radiation pressure, has been obscured by the much larger optically induced photothermal forces^46,47^. While the PVPZ effect is much stronger than radiation pressure effects, the role of photothermal forces must be carefully examined and managed. Our resonator is intentionally designed for use in its fundamental antisymmetric torsional mode to address this concern. The antisymmetric nature of the torsional oscillator design provides actuation and detection of only antisymmetric modes, with symmetric modes leading to cancellation. This cancellation is experimentally confirmed to first order by the two null experiments described above. The illumination wavelength is chosen such that the optical absorption within the GaAs layers is quite axially uniform, reducing photothermal forces. At this wavelength, the optical absorption is still sufficiently high to achieve good photovoltaic efficiency and minimize light transmission through the substrate, reducing another potential source of photothermal drive. Uniform optical illumination and absorption in a photothermal process would only drive symmetric modes since both tines would be actuated in the same way. Therefore, uniform photothermal processes would not actuate the antisymmetric mode or be detected by the antisymmetric piezoelectric detection. However, nonuniformities in optical actuation could in principle weakly drive the antisymmetric mode photothermally. We estimate the deflection from differential thermal expansion in the z-direction as would be expected from a fully nonuniform photothermal force (i.e., only illuminating a single tine) and find that it is 150 times smaller than the PVPZ effect (Supplementary Section XII) for a single tine. However, due to the nulling from the antisymmetric device design, the observed photothermal actuation would be substantially smaller, indicating that photothermal actuation likely makes a negligible contribution to the observed results. Additionally, the observed currents are in good agreement with expected values based on known and calibrated piezoelectric and photovoltaic effects. We also find (not shown) that the current upon actuation is modified by the bias voltage, which would only be expected for the PVPZ effect^40^.

In conclusion, we have demonstrated the PVPZ effect in a single-crystal GaAs micromechanical torsional resonator photodiode. Observation of this effect in GaAs utilizes its optical, mechanical, piezoelectric and semiconductor properties, coupling them through a device that was intentionally designed to demonstrate the PVPZ effect. The novel torsional resonator design allowed for planar antisymmetric actuation and a high quality factor, exploiting the anisotropic piezoelectric properties of GaAs while minimizing photothermal effects. However, the PVPZ effect could be harnessed in a wide variety of other optomechanical designs, including simple cantilever structures. The device was fabricated using conventional growth and fabrication technology, which allows integration with other device technologies. The PVPZ effect can be utilized for fast, strong optical actuation based on the intrinsic properties of GaAs with known design rules. In addition to this multidomain coupling, the PVPZ effect leads to a larger deflection than competing optical actuation mechanisms. We note that while we have measured the resonator deflection electrically, there is no requirement to have sample leads for the purpose of optical actuation alone. The multidomain coupling between the optical, mechanical and electrical DOFs can be exploited to develop novel devices and capabilities in integrated optoelectromechanical systems. For example, in optical computing, configurable optical circuits can be used to build optical switches^48^ and post-Moore or “More than Moore” technologies such as neuromorphic photonic chips^49^, systems for processing of big data with deep learning^50^ and quantum circuits^51,52^. With the success of measuring quantized motions of mechanical structures^53–55^, the PVPZ effect could be another approach to excite phonons and enable bidirectional^56,57^ quantum information transfer between the optical and electrical domains. The PVPZ effect can also be utilized in other III–V piezoelectric device platforms, such as InP and GaN. The former is of special interest as the prevailing platform for optical communication devices, while the latter platform is now widely used in the manufacture of white light solid-state sources and transistors for high-power electronics.

## Materials and methods

### Fabrication

A detailed description of the torsional resonator fabrication steps is given in Supplementary Section I. In brief, the torsional resonator geometry is first defined via wet etching, followed by formation of the bottom and top electrical contact pads and then ending with a timed etch release step. There are a total of six process steps consisting of four lithography steps, a back-contact step and a resonator release step. A single chip consists of 326 resonators and is 14 mm × 14 mm. The devices are isolated from each other by wet etching of the heterostructure layers between the devices. The impetus for this step is to minimize stray and feedthrough capacitances and resistances between devices.

## Supplementary information

Supplementary Material for Optical actuation of a micromechanical photodiode via the photovoltaic-piezoelectric effect
