# Supplementary material for: Optical actuation of a micromechanical photodiode via the photovoltaic-piezoelectric effect
Source: Microsyst Nanoeng. 2021 Apr 14;7:29. doi: 10.1038/s41378-021-00249-y (PMC8433330; doi:10.1038/s41378-021-00249-y)
Supplement: Supplementary file 1 — Supplementary Material for Optical actuation of a micromechanical photodiode via the photovoltaic-piezoelectric effect [file 41378_2021_249_MOESM1_ESM.docx]

**Supplementary Information**

**Optical actuation of a micromechanical photodiode via the photovoltaic-piezoelectric effect**

A. Rampal^1*†^, R. N. Kleiman^1^

1. **Fabrication**

The fabrication of the torsional resonator is a 6-step process consisting of 4 masking steps, a back-contact step and a resonator release step. A summary of the process steps follows. The fabrication begins with cleaving a 3” wafer with an MBE-grown heterostructure into 14 mm × 14 mm chips. Each chip contains 326 devices. The devices are isolated from each other by wet etching the heterostructure layers between the devices. The impetus for this step is to minimize stray and feed-through capacitances and resistances between devices. The devices, however, do share the same substrate. The fabrication begins by first cleaning the chips using the following recipe: 5-minute dip each in acetone and methanol followed by a 5-minute de-ionized H_2_O rinse. The chips are then placed in a UV-Ozone environment for 10 minutes followed by a 1-minute Buffered HF (BHF) dip and a 5-minute deionized (DI) water rinse. A review of the fabrication steps follows:

1. Mask #1 (Figure S1 (d)) defines the torsional resonator and its anchor. Using this mask, a 100 nm CVD-deposited SiO_2_ layer is patterned using photolithography and wet chemical etching using BHF. The exposed GaAs and InGaP layers are etched using C_6_H_8_0_7_:H_2_O_2_ (1:7) and HCl; respectively. The etching stops at the p^+^-GaAs layer.
2. Mask #2 (Figure S1 (e)) isolates the devices and opens up etch holes for the resonator’s release. The remaining SiO_2_ from the previous step is etched and a new 100 nm SiO_2_ layer is deposited. Using this mask, the SiO_2_ layer is patterned and etched. The exposed p^+^-GaAs layers are removed, isolating the devices and exposing the sacrificial Al_0.8_Ga_0.2_As layer.
3. Masks #3 and #4 (Figure S1 (f) and (g)) define the area for evaporation of metal on the bottom and top electrical contacts, i.e. on the p^+^ and n^+^-GaAs layers. For the p^+^ contact, Mask #3 is used. The SiO_2_ layer is patterned and etched using BHF. In this step, the developed photoresist is not removed. For the p^+^ contacts 25/50/200 nm of Ti/Pt/Au is deposited via e-beam evaporation in ultra-high vacuum. The sample is placed in an acetone bath for 24 hrs for lift-off. For the n^+^ contact, Mask #4 is used and follows the same steps as above. The only difference is the deposited metals are Ni/Ge/Au with thicknesses of 25/50/200 nm.
4. Following the masking steps, a metal back-contact is deposited on the substrate (Figure S1 (h)). The deposited metals are 25/50/200 nm of Ti/Pt/Au.
5. The resonators are released (Figure S1 (i)) in a wet etchant bath of (49%) HF:H_2_O (1:9) followed by a 10-minute DI rinse. The chip is then immersed in propanol and transferred to a critical point dryer (CPD) for release.

a


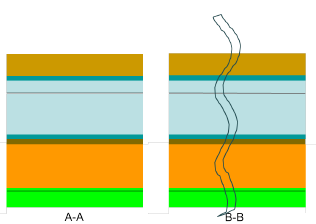


b

c

d


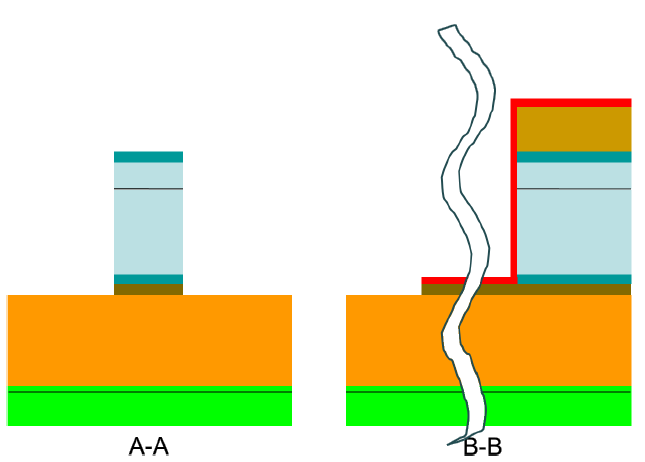


e


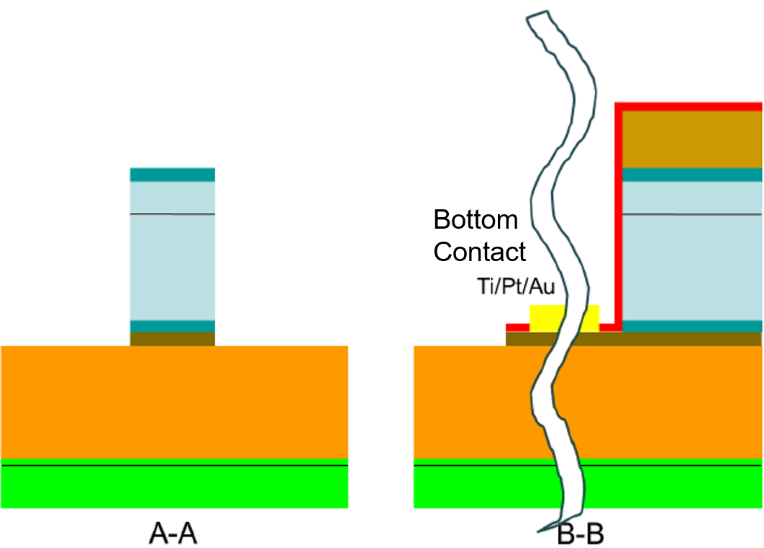


f


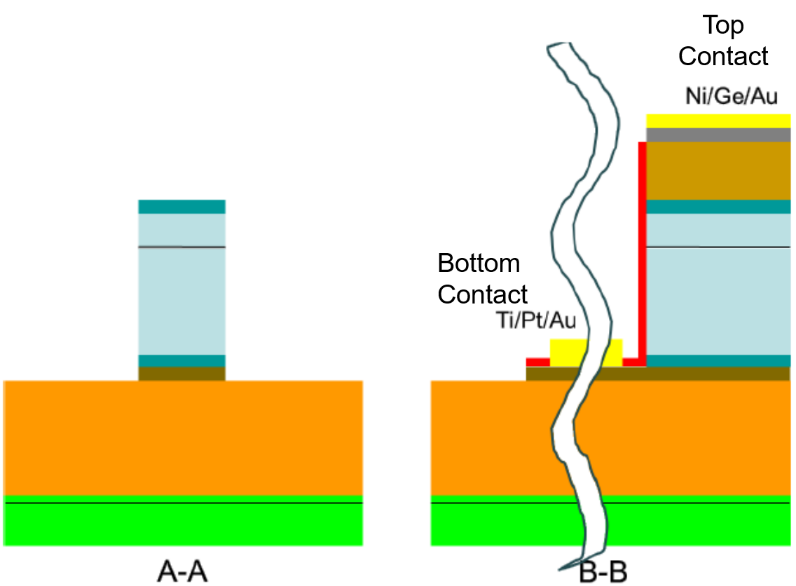


g


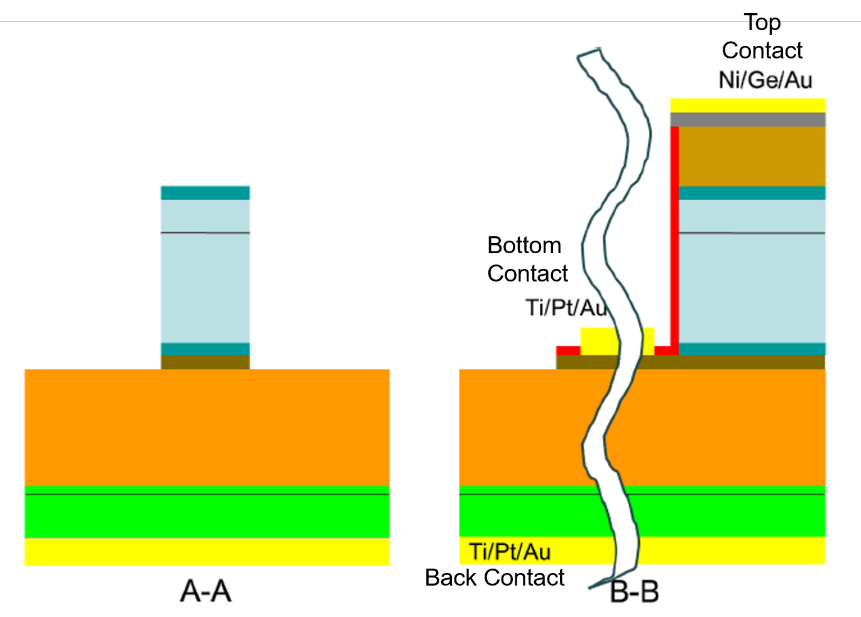


h


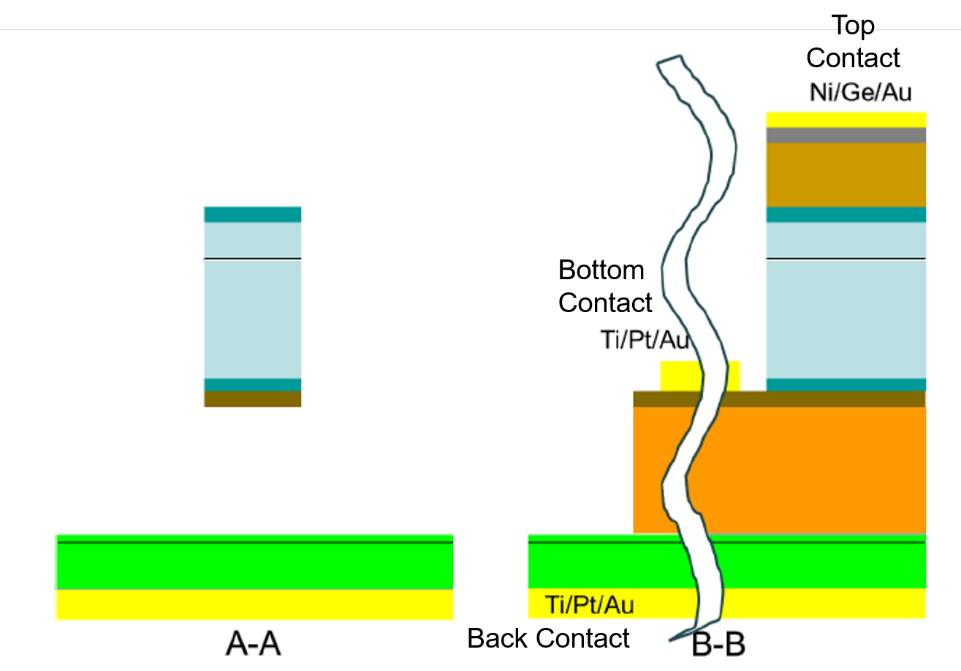


i

**Figure S1| Steps for fabricating the photodiode resonator.** **a**, Optical Micrograph of the fabricated photodiode resonator where cross-section A and B are the tine, top and bottom contacts. **b**, Cross-section views. Heterostructure cross-section at section A-A and B-B. The squiggly line in B-B separates the bottom and top contacts. **c**, Legend of the MBE heterostructure layers and additional layers used for fabrication. **d**, Mask #1 patterning of resonator and the anchor. **e**, Mask #2 isolation of devices by etching of p^+^ GaAs layer and opening of etch holes for release of resonator. **f**, Mask #3 evaporation of metal for bottom contacts. **g**, Mask #4 evaporation of metal for top contact. **h**, Back contact. Evaporation of metal for back contact. **i**, Release step. The sacrificial layer is etched to release the resonator

1. **Mounting the device for measurements**

The fabricated chip is mounted on a printed circuit board (PCB) using silver paste. A wire bonder is used to bond gold wires from the top and bottom contacts of the device to the electrical pads on the PCB. The PCB consists of 13 electrical pads connected to their respective SMA connectors via a transmission line.

1. **Summary of the MBE grown layers**

**Table S1 | Summary of the MBE-grown layers, thickness and doping concentrations**

| Layer | Grown Material | Doping Type | Doping Concentration (cm^3^) | Thickness (nm) | Purpose |
| --- | --- | --- | --- | --- | --- |
| 8 | GaAs | n^+^ | 5.0 x 10^18^ | 200 | Top Contact |
| 7 | In_0.485_Ga_0.515_P | n^-^ | 2.0 x 10^18^ | 25 | Passivation |
| 6 | GaAs | n^-^ | 1.0 x 10^18^ | 100 | n-junction |
| 5 | GaAs | p^-^ | 1.9 x 10^17^ | 1500 | p-junction |
| 4 | In_0.485_Ga_0.515_P | p | 1.0 x 10^18^ | 50 | Passivation |
| 3 | GaAs | p^+^ | 2.0 x 10^19^ | 200 | Bottom Contact |
| 2 | Al_0.8_Ga_0.2_As | i | 1.5 x 10^15^ | 1000 | Sacrificial |
| 1 | GaAs | p^+^ | 1.9 x 10^18^ | 50 | Buffer |

1. **Width and Position of the Depletion Region**

The width of the depletion region, *t*, of a p-n junction is given by (*59*);

$t=\sqrt{\frac{2\varepsilon_{r}\varepsilon_{0}V_{bi}}{q}\left( \frac{1}{N_{a}}+\frac{1}{N_{d}} \right)}$ (S1)

where

$V_{bi}=\frac{kT}{q}ln\left( \frac{N_{a}N_{d}}{n_{i}^{2}} \right)=0.026ln\left( \frac{N_{a}N_{d}}{n_{i}^{2}} \right)$ (S2)

at room temperature. *N_a_* and *N_d_* are the doping concentrations of the p and n regions, given in Table S1, and *n_i_* is the intrinsic carrier concentration which for GaAs is 2.0 x 10^6^ cm^-3^. The relative permittivity, *ε_r_*, of GaAs is 13.18^60^. Therefore, for the GaAs p-n junction, *t* = 146.2 nm and *V_bi_* = 1.33 V.

We can also determine the extent of the depletion region into the p and n regions using (*59*) $x_{p0}=\frac{t}{1+\frac{N_{a}}{N_{d}}}$ and $x_{n0}=\frac{t}{1+\frac{N_{d}}{N_{a}}}$ and find that are: $x_{p0}=$132.90 and $x_{n0}=$13.29 nm, totaling *t* = 146.2 nm, as above. The undepleted p and n regions are 1367.10 nm and 86.71 nm respectively. These thicknesses are used to construct the functional layer thicknesses shown in Figure 1 and used in subsequent calculations.

The total area of the device includes the area of the pad and the area of the resonator. The dimensions of the pad are 250 x 250 μm^2^ and the area of the resonator is 7607.1 μm^2^. The capacitance of the p-n junction is: 55.0 pF and calculated using:

$C_{J}=\frac{\varepsilon_{r}\varepsilon_{0}A}{t}$ (S3)

where *A* is the combined area of the pad and resonator.

1. **Mode Shapes of the resonator**

Figure S1 (a) – (c) are the expected torsional mode shapes of the resonator. In the first mode, actuated and detected in this work, the pad and the tines move together with respect to the torsion axis, while in the second and third modes the pad and the tines move oppositely with respect to the torsion axis. The commonality amongst these three modes is that the tines exhibit antisymmetric flexural deformation with respect to the torsion axis resulting in torsional motion.

**a**

**b**

**c**

**Figure S2 | First three resonator torsional mode shapes.** a) In the first torsional mode the pad and the tines rotate in the same direction. The tines, similar to the fundamental mode of a cantilever, have no nodes and resemble the deflection of a cantilever. (b) and (c) are modes where the pad and the tines rotate in the opposite direction. The tines, similar to the 2^nd^ and 3^rd^ modes of a cantilever, have 1 and 2 nodes respectively. However, these modes also exhibit a complex flexure around the cantilever axis. The color scale indicates increasing strain perpendicular to the device plane from none (blue) to maximum (red) in dimensionless units.

1. **Current in the modified BVD Circuit**

The complex current, *i*, for the modified BVD circuit, Figure 2b, is:

$i=i_{re}+ji_{im}$ (S4)

where *i_re_* and *i_im_* are the real and imaginary parts of *i*. From circuit analysis these are given by:

$\begin{aligned} i_{re}=\frac{V_{ac}((\alpha^{2}R_{s}\gamma+\beta)\omega_{0}^{4}+(R_{s}C_{J}^{2}\gamma+\beta)\omega^{4})Q^{2}+Q\omega_{0}^{3}C_{m}\omega^{2}R_{sh}(R_{sh}+2R_{s})}{D}+ \\ \frac{V_{ac}((-2\alpha Q^{2}+C_{J})C_{J}R_{s}\gamma+(1-2Q^{2})R_{sh}-2R_{s}Q^{2}+R_{s})\omega^{2}\omega_{0}^{2}}{D} \end{aligned}$ (S5)

$i_{im}=\frac{-V_{ac}\omega R_{sh}^{2}(-\omega_{0}^{2}\omega^{2}C_{J}-Q^{2}(\omega_{0}^{4}(C_{m}-C_{J})+\omega_{0}^{2}\omega^{2}(2C_{J}+C_{m})-\omega^{4}C_{J}))}{D}$ (S6)

Where

$$D=((1+\alpha^{2}\gamma^{2})R_{s}^{2}+(2R_{s}+R_{sh})R_{sh})Q^{2}\omega_{0}^{4}+2\beta C_{m}R_{s}R_{sh}\omega^{2}Q\omega_{0}^{3}+((-2\alpha C_{J}\omega^{2}\gamma^{2}R_{s}^{2}-2\omega^{2}\beta^{2})Q^{2}+(R_{sh}^{2}C_{J}^{2}R_{s}^{2}+\beta^{2})\omega^{2})\omega_{0}^{2}+(C_{J}^{2}R_{sh}^{2}\omega^{2}R_{s}^{2}+\beta^{2})\omega^{4}Q^{2}$$

$$\begin{aligned} \omega_{0}=\frac{1}{\sqrt{L_{m}C_{m}}} \\ Q=\frac{1}{\omega_{r}R_{m}C_{m}} \\ \alpha=C_{m}+C_{J} \\ \beta=R_{s}+R_{sh} \\ \gamma=\omega^{2}R_{sh}^{2} \end{aligned}$$

1. **Analytic expression for the motional capacitance, *C_m_***

To find an analytic expression for the motional capacitance, we assume the torsional mode can be approximated by the fundamental flexural mode of a cantilever. The justification for this is that given the orientation of the tines with respect to the crystallographic orientations shown in Figure 1B, the cantilever-shaped tines are the only part of the structure that have a piezoelectric response that contributes to the torsional mode. Given the layer structure shown in Figure 1C, the expression for the motional capacitance is given by^32^:

$C_{m}=\frac{-6\beta LYd_{23}^{2}W{(t_{1}-t_{3})}^{2}}{t^{3}}$ (S7)

where $\beta=\frac{1}{\left( k_{i}L \right)^{2}}\left( \frac{\sin\left( k_{i}L \right)\sinh\left( k_{i}L \right)}{\sin\left( k_{i}L \right)+sinh\left( k_{i}L \right)} \right)^{2}$ and *k_i_L* are the constants^61^ for the cantilever flexural mode shape (*k_1_L* = 1.875, *k_2_L* = 4.694, …)*,* and *Y*, *d_23_, L* and *W* are modulus of elasticity, piezoelectric constant, length and width of the tines, respectively. The values for*Y*, *d_23_, L* and *W* are 80 GPa, 1.345 pm/V, 80.0 μm, and 30.0 μm. The total thickness, *t*, is the sum of *t_1_*_,_ *t_2_* and *t_3_*, the thickness below the depletion region, the thickness of the depletion region and the thickness above the depletion region, respectively. The values for *t_1_*_,_ *t_2_* and *t_3_*, are 1.604 μm, 146.2 nm and 125 nm, yielding a value of $C_{m}=0.43 fF$. This value is in reasonable agreement with experimental values determined from electrical (Section F) and optical actuation (Section H). equation (S7) accounts for the fact that the structure consists of two tines. The details for the derivation are given in^32^ but briefly the expression is derived using:

1. The piezoelectric charge $Q=\int_{0}^{L} \int_{0}^{W} d_{23}Y\varepsilon dydx$ where *Q* is the charge and $\varepsilon$ is the strain.
2. The layers comprising the bimorph are assumed to be thin enough that the radius of curvature, *r*, is the same across all layers $\frac{1}{r}=\frac{\sum_{j} M_{j}}{\sum_{j} YI_{j}}$; were *M_j_* is the moment of each of the layers and *I_j_* is moment inertia of the respective 3 layers.
3. The total strains at the interface of each of the layers must be equivalent to each other, i.e. $\epsilon_{1}\left( t_{1} \right)=\epsilon_{2}\left( t_{1} \right)\text{ }$ and $\epsilon_{2}(t_{2})=\epsilon_{3}(t_{2})$.
4. **Circuit parameters from fitting the electrical actuation data**

The current vs. frequency data shown in Figure 2c is fit to equation (S4), based on the equivalent circuit shown in Figure 2b. The resulting fit parameters are given in Table S2.

The value of *C_J_* corresponds to a square pad with dimensions of 250 x 250 μm^2^ spaced by a depletion width and is in reasonable agreement with the value expected from evaluation of equation (S3). The value for *R_s_* is expected to be 0.14 Ω (as calculated from the doping concentration, given in Table S1, of the top and bottom contacts) indicating additional sources of series resistance. The fitted value of *C_m_* is close to the value predicted in Sections E.

**Table S2 | Circuit parameters for equivalent circuit in Figure 2b**

| Parameter | Symbol | Value |
| --- | --- | --- |
| Motional Resistance (kΩ) | *R_m_* | 386 |
| Motional Capacitance (*f*F) | *C_m_* | 0.49 |
| _­_Motional Inductance (kH) | *L_m_* | 8.75 |
| Junction Capacitance (pF) | *C_J_* | 38.1 |
| Resonant Frequency (Hz) | *f_0_* | 76,696 |
| Quality Factor | *Q* | 10,116 |
| Shunt Resistance (GΩ) | *R_sh_* | 1.3 |
| Series Resistance (Ω) | *R_s_* |  |

1. **Photodiode characterization with DC illumination**

Figure S4 is an illustration of the setup used to measure the I-V curves shown in Figure 3c. The equation for the DC current is given by^36^:

$I=I_{th}[\exp(\alpha(V-IR_{s}))-1]+\frac{V-IR_{s}}{R_{sh}}-I_{sc}$ (S8)

where *V* is the applied voltage, *I_sc_* is the short circuit current, *I_th_* is the thermal current and $\alpha=\frac{q}{nk_{B}T}$ where *q*, *k_B_*, *T* and *n* are the charge, Boltzmann constant, temperature and diode ideality factor. The values of *R_sh_*, *R_s_* and *n* are determined by fitting equation (S8) to the measured current. The series resistance values are much higher than those found from electrical actuation. In the electrical actuation, the measurements are performed at V_DC_ = 0, while the fit for the series resistance in the I-V curves is dominated by the characteristics near V_oc_. We hypothesize that there is an additional parasitic junction associated with the lower In_0.485_Ga_0.515_P passivation layer (#4 in Table S1) that is depleted with increasing bias voltage, increasing the series resistance.

**Table S3 | Fitted values for *R_sh_*, *R_s_* and *n* for different incident optical powers**

| P_opt_ (μW) | R_sh_ (GΩ) | R_s_ (kΩ) | *n* |
| --- | --- | --- | --- |
| 0.304 | 1.0 | 19.00 | 1.93 |
| 0.399 | 1.0 | 17.85 | 2.11 |
| 1.614 | 1.0 | 13.98 | 2.64 |

1. **Circuit parameters from fitting the optical actuation data**

The current vs. frequency data shown in Figure 4d is fit to equation (3), based on the equivalent circuit shown in Figure 4c. The resulting fit parameters are given in Table S4. Considering the different operating conditions between electrical and optical actuation, the fit parameters are reasonably consistent with those found for electrical actuation, as given in Table S2.

**Table S4 |Circuit parameters for equivalent circuit in Figure 4c**

| Parameter | Symbol | 1.61 nW | 3.34 nW | 17.6 nW |
| --- | --- | --- | --- | --- |
| AC optical power (nW) | *p_opt_* | 1.61 | 3.34 | 17.6 |
| Motional Resistance (kΩ) | *R_m_* | 345 | 407 | 434 |
| Motional Capacitance (*f*F) | *C_m_* | 0.875 | 0.875 | 0.850 |
| ­Motional Inductance (kH) | *L_m_* | 4.92 | 4.92 | 5.07 |
| Junction Capacitance (pF) | *C_J_* | 55.0 | 55.0 | 55.0 |
| Resonant Frequency (Hz) | *f_0_* | 76,707 | 76,707 | 76,667 |
| Quality Factor | *Q* | 6,875 | 5,825 | 5,625 |
| Shunt Resistance (GΩ) | *R_sh_* | 1.0 | 1.0 | 1.0 |
| Series Resistance (kΩ) | *R_s_* | 3.0 | 3.2 | 3.6 |

1. **IR LED Power**

Figure S3 is a plot of the measured DC LED power, *P_opt_* incident on the opto-piezo resonator. The x-axis is the LED bias voltage, and the y-axis is the LED power measured using the 818-BB-20 diode and the 1815-C Newport Power Meter, after area scaling. The uncertainty in the measured power, *P_opt_*, is calculated from the table given in the operator's manual, which reports the maximum error is 1.7%. The LED is placed 0.5 cm away from the photodiode, matching the experimental configuration where the LED is placed 0.5 cm away from the opto-piezo resonator. The power on the opto-piezo resonator is determined by scaling its illuminated area of 2.1x10^-8^ m^2^ to the illuminated area of the 818-BB-20, which is 1.3x10^-7^ m^2^.

**Figure S3 | LED Power.** LED illuminated power on the opto-piezo resonator, *P_opt_* measured using 818-BB-20 at a distance of 0.5 cm

1. **Photothermal Cantilever Deflection**

We have simulated the thermally driven deflection of a cantilever, using Mechanical APDL (ANSYS Parametric Design Language), with the following expression for the thermal gradient:

$\Delta T(z)=\frac{p_{opt}}{\kappa\gamma A}(e^{-\gamma z}-e^{-\gamma t})$ (S9)

where *A = LW* is the surface area of the cantilever with *L* and *W* being 80 and 30 μm respectively. The total thickness, *t* of the cantilever is 1.875 μm. For GaAs, the thermal expansion coefficient, *α*, and the thermal conductivity, $\kappa$, are 5.73 X 10^-6^/K and 55 W/(m-K) respectively. The variable z is the distance from the light-impinging surface into the substrate. equation (S9) is the solution to the 1-dimensional heat equation in the presence of optical absorption, with the optical absorption constant, $\gamma$, taken as 1.0 X 10^4^ cm^-1^ for GaAs. From the simulation we find a thermal deflection at the cantilever tip of $\delta_{z}\sim67{nm}/W$. For the experimental conditions ($p_{opt}=17.6 nW$ and *Q* = 5625) of PVPZ actuation (Figure 4e) the thermal deflection of a single tine is $\delta_{z}=7.20 pm$, which is ~150 times smaller than the PVPZ deflection of 1.1 nm. However, based on this model, thermal deflection would drive both tines in the same direction and not actuate the antisymmetric mode being measured.

**III. References**

59) Sze, S. M., *Physics of Semiconductor Devices* (Wiley-Interscience, third edition 1981). <https://doi.org/10.1002/0470068329>

60) Adachi, S., GaAs, AlAs, and Al_x_Ga_1−x_As: Material parameters for use in research and device applications, *J. Appl. Phys.* **58,** R1 (1985). <https://doi.org/10.1063/1.336070>

61) Weaver Jr. W., Timoshenko, S. P., Young, D. H. *Vibrations Problem in Engineering*, (Wiley-Interscience, 1990)
